# International trends in lung cancer incidence from 1973 to 2007

**DOI:** 10.1002/cam4.1359

**Published:** 2018-03-14

**Authors:** Yue Zhang, Jian‐Song Ren, Hui‐Yao Huang, Ju‐Fang Shi, Ni Li, Yawei Zhang, Min Dai

**Affiliations:** ^1^ National Cancer Center / Cancer Hospital Chinese Academy of Medical Sciences and Peking Union Medical College Beijing 100021 China; ^2^ Beijing Obstetrics and Gynecology Hospital Capital Medical University Beijing 100026 China; ^3^ Yale School of Medicine Yale Cancer Center New Haven Connecticut

**Keywords:** Average annual percentage change, incidence, international trends, joinpoint analysis, lung neoplasms

## Abstract

Lung cancer is the commonly diagnosed cancer and one of the most important avoidable causes of death around the world. We conducted the study to investigate the pattern of lung cancer incidence worldwide. Joinpoint analysis was used to extend international lung cancer incidence rates by the latest data from Cancer Incidence in Five Continents over the 35‐year period 1973–2007 from 24 populations from Americas, Asia, Europe, and Oceania. Age‐standardized incidence rates (ASRs) of lung cancer were from 33.3 to 66.8 per 100,000 among males and 10.5 to 37.4 per 100,000 among females in most of Americas, Europe, and Oceania populations during the period 2003–2007. In Asia, ASRs in China (Hong Kong) were the highest, up to 53.3 per 100,000 in males and 21.9 per 100,000 in females during the period 2003–2007. The international trends between 1973 and 2007 showed that ASRs of lung cancer among males were declining in 13 of 18 selected Americas, Oceania, and Europe populations, with AAPC from −0.7% to −2.9%, whereas the rates among females in 18 selected populations were increasing, with AAPC from 1.3% to 5.0%. The increasing and decreasing trends of ASRs of lung cancer in Asia have a geographic variation but no gender differences. Although the decreasing trends in ASRs of lung cancer for males were observed, the ASRs were higher than females. The declining trends in males were mainly attributed to tobacco control, whereas the increasing trends in females should be given more concern and need to be further studied in etiology factors.

## Introduction

Lung cancer is the commonly diagnosed cancer and one of the most important avoidable causes of death around the world. According to GLOBOCAN 2012, lung cancer is estimated to cause 1.6 million deaths in the world, which were quantitatively close to the corresponding 1.8 million cases 1. Lung cancer is more common in males than females with a cumulative life time (0–74) risk of 4% and 1%, respectively 1. Whereas the incidence rates for males have been declining in most areas of North America, the Nordic countries, Europe, and Oceania, while rates for females have been rising rapidly virtually everywhere 2. Tobacco smoking may be attributable to lung cancer pattern. Blot reported that the majority (85–90%) of lung cancer among women like in men are considered to be caused by smoking 3.

The major histological subtypes of lung cancer comprise adenocarcinoma, squamous carcinoma, small cell carcinoma, and large cell carcinoma, which in aggregate, account for approximately 99% cases of primary lung cancer 4. The predominant form of lung cancer has been squamous carcinoma in males and adenocarcinoma in females, although adenocarcinoma has surpassed squamous cell carcinoma in frequency among males in several populations in recent years 2.

Some studies have described the international lung cancer trends by histologic subtypes except for Asia 2, 5, 6. The patterns and trends in lung cancer incidence have varied because of difference in smoking patterns and exposures to other lung carcinogens 7, 8. Herein, we examined the international trends in overall lung cancer incidence among males and females in 24 selected populations from Americas, Asia, European, and Oceania during 35‐year period (1973–2007) and described the distribution of lung cancer incidence by histological subtypes based on the 2003–2007 incidence data.

## Materials and Methods

### Incidence data

To examine the changing trends in the incidence of lung cancer over time, age‐standardized (by world standard population 9) incidence rates (ASRs) by sex were obtained from Volumes 4–10 of Cancer Incidence in Five Continents (CI5) 10, 11, 12, 13, 14, 15, 16 from the Web site of International Agency for Research on Cancer (IARC). Volumes 4–10 of CI5 generally provided data by 5‐year period: 1973–1977, 1978–1982, 1983–1987, 1988–1992, 1993–1997, 1998–2002, and 2003–2007. Incidence data by histologic subtypes (adenocarcinoma, squamous carcinoma, small cell carcinoma, and large cell carcinoma) from 2003 to 2007 were collected from 24 populations in four continents from Vol. 10 of CI5. Classification of lung cancer from Vols. 4, 5–8, and 9–10 of CI5 was coded according to the International Classification of Diseases (ICD) 8th (163), 9th (163), and 10th (C33‐C34) revisions, respectively.

Populations were chosen for inclusion in our study on the basis of the following criteria: (1) incidence for time periods at least as far back as 1983–1987; (2) no changes in population coverage or warnings regarding data quality reported in CI5 Vols. 4–10; and (3) a sufficiently large number of registered cases in CI5 Vol. 10 to enable analyses of recent rates by histologic subtypes (trends by histologic subtypes were not included in our study). Only one registry from each country was selected; if more than one registry met the basic criteria, the registry with the largest population was included in the analysis (except for China, Hong Kong, and Shanghai). A total of 24 populations were selected: four from the Americas (Canada, British Columbia (BC); Colombia, Cali; USA, Black/White), six from Asia (China, Hong Kong; China, Shanghai; India, Mumbai; Israel, Jews; Japan, Osaka Prefecture; Singapore, Chinese), five from Northern Europe (Denmark; Finland; Norway; Sweden; UK, England, North Western Region (NWR)), three from Western Europe (France, Bas‐Rhin; Germany, Saarland; Switzerland, Geneva), four from elsewhere in Europe 17 (Southern and Central & Eastern Europe including Italy, Varese Province; Poland, Cracow; Slovakia; Spain, Navarra), and two from Oceania (Australia, New South Wales (NSW); New Zealand). No African populations met all the inclusion criteria. However, four African populations (Algeria, Setif Wilaya; Egypt, Gharbiah; Uganda, Kyadondo; Zimbabwe, Harare: African) were chosen to describe the lung incidence in the last time interval (2003–2007).

Incidence data for white and black populations in United States were not included in CI5 vols. 4 (1973–1977) and 5 (1978–1982), so we further referred to the U.S. Surveillance Epidemiology and End Results (SEER) dataset 18. SEER program is a population‐based cancer registration system covering 18 registries and 28% of the U.S. population. Long‐term data from 1973 to 2010 were available from nine registries that included approximately 9.4% of the U.S. population (based on 2010 census).

The data for New Zealand between 1983–1987 and 1988–2002 were not included in CI5, but contained in CI5*plus* 19 which is a database of successive volumes of CI5 series. So we abstracted the data for New Zealand during the periods 1983–1987 and 1988–2002 from CI5*plus* and the latest time period 2003–2007 from CI5 vol.10.

### Data analysis

Incidence trends in ASRs of lung cancer were analyzed using Joinpoint regression (Joinpoint regression software, version 3.5.3‐May 2012, available through the Surveillance Research Program of the US National Cancer Institute). The permutation method was used for significance tests. Changes in annual incidence rates from lung cancer were calculated as annual percentage change (APC) in each segment. In the final model, the Joinpoint analysis provided average annual percentage change (AAPC). The significant test of APC and AAPC to 0 was also conducted.

Age‐standardized incidence rates of lung cancer by histologic subtypes (adenocarcinoma, squamous carcinoma, small cell carcinoma, and large cell carcinoma) and sex for selected populations during the period 2003–2007 were integrated and calculated. Secular trends in ASRs of lung cancer were examined by registries, sexes for every five‐year period during 1973–2007. The trends in ASRs of lung cancer from New Zealand were described during five‐year time periods from 1983–1987 to 2003–2007. Figures displaying the incidence trends were prepared using a semilog scale to facilitate the comparison of temporal trends as well as magnitude. These data were plotted at the midpoint of each five‐year interval.

## Results

Age‐standardized incidence rates of lung cancer in 2003–2007 were the highest in Americas and Europe (U.S. black, Poland, and Germany) and much lower in most of populations in Asia and Africa (India, Algeria, and Uganda; Tables 1 and 2). In most of Americas, Europe, and Oceania, ASRs of lung cancer ranged from 33.3 to 66.8 per 100,000 among males and 10.5 to 37.4 per 100,000 among females other than Colombia (19.0 per 100,000 in males and 8.9 per 100,000 in females, respectively). In Asia, ASRs in China (Hong Kong) were the highest, up to 53.3 per 100,000 in males and 21.9 per 100,000 in females. The variation of ASRs ranged from 29.8 per 100,000 in Israel (Jews) to 44.7 per 100,000 in Singapore (Chinese) among males and from 13.4 per 100,000 in Israel (Jews) to 18.1 per 100,000 in China (Shanghai) among females. In Africa, the extent of ASRs was between 10.1 per 100,000 and 19.9 per 100,000 in males and from 3.7 per 100,000 to 6.4 per 100,000 in females.

**Table 1:** International variation in lung cancer incidence rates, from 1973–1977 to 2003–2007

Country	Period of registry established	Mean of MV%^a^	Males				Joinpoint analyses (1973–2007)				
			1973–1977		2003–2007		Trend 1		Trend 2		AAPC (%)
			Cases	Rate^b^	Cases	Rate^b^	Period	APC (%)	Period	APC (%)	
Northern Europe											
Denmark	1953–1957	86.2	7585	51.8	10,822	45.0	1975~1985	0.9	1985~2005	−1.3^g^	−0.7^g^
			(1973–1976)								
Finland	1959–1961	85.6	11,523	74.4	8126	34.4	1975~1985	−1.6	1985~2005	−3.4^g^	−2.9^g^
			(1971–1976)								
Norway	1959–1961	89.6	3710	25.4	7182	36.7	1975~2005	1.0^g^			1.0^g^
Sweden	1959–1961	97.9	8007	23.8	9087	20.7	1975~2005	0.4	1985~2005	−1.1^g^	−0.7^g^
			(1971–1975)								
UK, England, NWR	1973–1977	53.8	12,807	86.0	14,188	47.4	1975~1985	−0.6	1985~2005	−2.8^g^	−2.2^g^
Western Europe											
France, Bas‐Rhin	1975–1977	92.1	860	54.3	2098	53.2	1975~1990	1.5^g^	1990~2005	−1.7^g^	−0.1
			(1975–1977)								
Germany, Saarland	1968–1972	79.0^c^	2629	74.4	3048	58.4	1975~1990	0.5	1990~2005	−1.2^g^	−0.9^g^
Switzerland, Geneva	1970–1972	95.1	703	69.4	743	42.9	1975~2005	−1.8^g^			−1.8^g^
Europe, Other											
Italy, Varese Province	1976–1977	73.6	642	70.4	2170	51.3	1975~1985	1.9	1985~2005	−2.4^g^	−1.2^g^
			(1976–1977)								
Poland, Cracow	1973‐1977	66.1	1138	74.6	1252	58.7	1975~2005	−0.8^g^			−0.8^g^
					(2003–2006)						
Slovakia	1973–1977	70.9	2549	43.8	9453	56.2	1975~1985	6.4^g^	1985~2005	−2.1^g^	0.3^g^
Spain, Navarra	1973–1977	84.8	357	23.5	1350	50.5	1975~2005	2.3^g^			2.3^g^
Americas											
Canada, BC	1969–1972	78.1	3501	49.0	7097	38.3	1975~1985	2.1	1985~2005	−2.3^g^	−0.8^g^
Colombia, Cali	1967–1971	53.9	193	19.5	810	19.0	1975~1985	2.4	1985~2005	−1.5^g^	−0.4
			(1972–1976)								
USA, SEER: Black	1973–1975	91.8^d^	3043^e^	87.1	4972	66.8	1975~1985	2.2^g^	1985~2005	−2.4^g^	−0.9^g^
USA, SEER: White	1973–1975	90.7^d^	28,365^e^	60.4	35,816	44.8	1975~1985	0.8	1985~2005	−1.9^g^	−1.1^g^
Oceania											
Australia, NSW	1973–1977	82.1	6756	51.4	9665	34.7	1975~1985	0.1	1985~2005	−2.1^g^	−1.5^g^
New Zealand	1960‐1962	75.7^f^	–	–	5124	33.3	1985~2005	−2.6^g^			−2.6^g^
Asia											
China, Hong Kong	1974–1977	63.5	3666	55.5	14,003	53.3	1975~1985	4.0^g^	1985~2005	−1.9^g^	−0.2
			(1974–1977)								
China, Shanghai	1975	44.0	1306	51.2	12,413	40.9	1975~2005	−0.7			−0.7
			(1975)								
India, Mumbai	1964–1966	60.0	714	14.2	2134	9.3	1975~2005	−1.7^g^			−1.7^g^
			(1973–1975)								
Israel: Jews	1960–1966	81.7	2254	29.3	5114	29.8	1975~2005	0.2^g^			0.2^g^
			(1972–1976)								
Japan, Osaka Prefecture	1970‐1971	70.9	4713	29.4	19,929	41.6	1975~1985	3.8^g^	1985~2005	0.0	1.0^g^
Singapore: Chinese	1950‐1961	79.6	1734	68.0	3338	44.7	1975~1985	0.2	1985~2005	−2.2^g^	−1.5^g^
Africa											
Algeria, Setif Wilaya	1986–1989		–	–	466	19.9					
Egypt, Gharbiah	1999‐2002		–	–	795	11.9					
Uganda, Kyadondo	1954‐1960		–	–	59	5.2					
Zimbabwe, Harare: African	1990‐1992		–	–	87	10.1					
					(2003–2006)						

APC, annual percent change; AAPC, average annual percent change.

a Average of MV% (Percentage of morphologically verified cases) was calculated from 1978 to 2007.

b Rate is age‐standardized to the world population, per 100,000 person‐years.

c Germany, Saarland (1983–2007).

d USA, SEER: Black/White (1988–2007).

e The data of USA, SEER: Black/White were from SEER nine registries database.

f New Zealand (1993–2007).

g APC/AAPC is significantly different from 0 (two‐sided *P* < 0.05).

**Table 2:** International variation in lung cancer incidence rates, from 1973–1977 to 2003–2007

Country	Period of registry established	Mean of MV%^a^	Females				Joinpoint analyses (1973–2007)				
			1973–1977		2003–2007		Trend 1		Trend 2		AAPC (%)
			Cases	Rate^b^	Cases	Rate^b^	Period	APC (%)	Period	APC (%)	
Northern Europe											
Denmark	1953–1957	86.5	1967	12.1	9252	35.2	1975~1985	6.7^g^	1985~2005	2.1^g^	3.4^g^
			(1973–1976)								
Finland	1959–1961	85.0	1245	5.8	3250	10.8	1975~1985	2.5^g^	1985~2005	1.7^g^	1.9^g^
			(1971–1976)								
Norway	1959–1961	89.0	881	5.4	4886	23.3	1975~1995	5.7^g^	1995~2005	3.3^g^	5.0^g^
Sweden	1959–1961	97.6	2161	5.7	7791	17.2	1975~1985	5.2^g^	1985~2005	2.9^g^	3.6^g^
			(1971–1975)								
UK, England, NWR	1973–1977	51.1	3194	16.8	11,280	30.8	1975~1985	4.5^g^	1985~2005	0.7	1.8^g^
Western Europe											
France, Bas‐Rhin	1975–1977	89.5	94	4.2	670	14.5	1975~1985	3.2^g^	1985~2005	5.1^g^	4.6^g^
			(1975–1977)								
Germany, Saarland	1968–1972	76.8^c^	276	5.4	1215	21.1	1975~1985	3.4^g^	1985~2005	5.3^g^	4.7^g^
Switzerland, Geneva	1970–1972	93.1	121	8.5	405	18.7	1975~2005	2.6^g^			2.6^g^
Europe, Other											
Italy, Varese Province	1976–1977	66.1	71	5.8	646	12.6	1975~1985	3.3^g^	1985~2005	2.4^g^	2.6^g^
			(1976–1977)								
Poland, Cracow	1973‐1977	60.7	269	11.1	568	17.9	1975~1995	2.2^g^	1995~2005	0.4	1.7^g^
					(2003–2006)						
Slovakia	1973–1977	63.4	309	4.4	2465	10.5	1975~2005	2.2^g^			2.2^g^
Spain, Navarra	1973–1977	75.9	49	2.6	232	8.9	1975~2005	3.6^g^			3.6^g^
Americas											
Canada, BC	1969–1972	78.9	1054	14.1	6440	32.1	1975~1985	7.3^g^	1985~2005	0.7	2.5^g^
Colombia, Cali	1967–1971	55.0	71	5.4	512	8.9	1975~1985	5.7^g^	1985~2005	−0.8^g^	1.3^g^
			(1972–1976)								
USA, SEER: Black	1973–1975	90.6^d^	852^e^	20.4	3919	37.4	1975~1985	4.2^g^	1985~2005	−0.2	2.0^g^
USA, SEER: White	1973–1975	89.7^d^	10,068^e^	18.4	33,168	34.6	1975~1985	5.5^g^	1985~2005	0.6^g^	2.0^g^
Oceania											
Australia, NSW	1973–1977	81.6	1343	8.7	5824	19.2	1975~1985	4.5^g^	1985~2005	1.7^g^	2.5^g^
New Zealand	1960‐1962	74.7^f^	–	–	3873	23.5	1985~2005	1.3^g^			1.3^g^
Asia											
China, Hong Kong	1974–1977	57.6	1942	23.4	6817	21.9	1975~1985	3.7^g^	1985~2005	−1.9^g^	−0.3
			(1974–1977)								
China, Shanghai	1975	39.0	539	18.1	6238	18.1	1975~2005	0.1			0.1
			(1975)								
India, Mumbai	1964–1966	57.6	154	4.0	824	3.5	1975~2005	−0.3			−0.3
			(1973–1975)								
Israel: Jews	1960–1966	79.8	725	9.0	2887	13.4	1975~2005	1.4^g^			
			(1972–1976)								
Japan, Osaka Prefecture	1970‐1971	64.5	1748	8.5	8408	13.9	1975~1985	3.3^g^	1985~2005	0.9	1.6^g^
Singapore: Chinese	1950‐1961	74.5	624	19.8	1713	17.6	1975~2005	−0.7^g^			−0.7^g^
Africa											
Algeria, Setif Wilaya	1986–1989		–	–	69	2.6					
Egypt, Gharbiah	1999‐2002		–	–	257	3.7					
Uganda, Kyadondo	1954‐1960		–	–	55	5.1					
Zimbabwe, Harare: African	1990‐1992		–	–	49	6.4					
					(2003–2006)						

APC, annual percent change; AAPC, average annual percent change.

a Average of MV% (Percentage of morphologically verified cases) was calculated from 1978 to 2007.

b Rate is age‐standardized to the world population, per 100,000 person‐years.

c Germany, Saarland (1983–2007).

d USA, SEER: Black/White (1988–2007).

e The data of USA, SEER: Black/White were from SEER nine registries database.

f New Zealand (1993–2007).

g APC/AAPC is significantly different from 0 (two‐sided *P* < 0.05).

**Figure 1:**
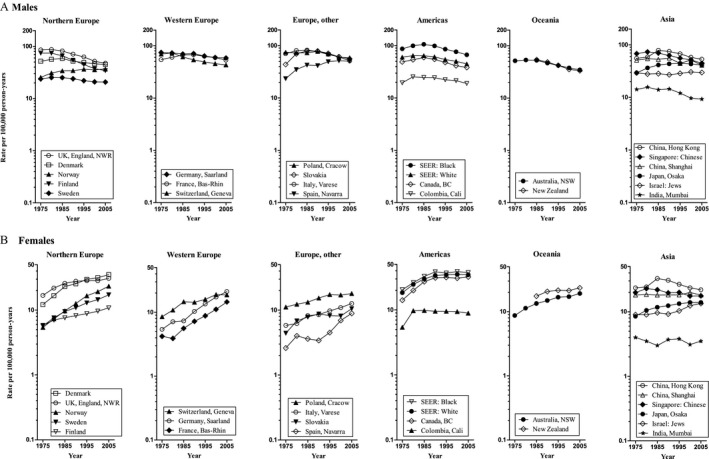
Trends in age‐standardized lung cancer incidence rates by continent and area for the time period 1973–2007: (A) Males, (B) Females.

In American, European, and Oceania populations, there were different trends in ASRs for lung cancer for both males and females. Among males, ASRs for lung cancer in 13 of 18 populations decreased significantly from 1973 to 2007, with AAPC from −0.7% to −2.9% (*P* < 0.05; Table 1 and Fig. 1A), of which nine populations including Denmark, Finland, Sweden, UK, England, Germany, Italy, Canada, USA: Black, USA: White and Australia kept declining after mid‐1990s. In contrast, ASRs for lung cancer in Norway, Slovakia, and Spain showed a significant increase trends from 1973 to 2007, with AAPC of 1.0%, 0.3%, and 2.3% (*P* < 0.05), respectively. ASRs for lung cancer in France and Colombia leveled off in the whole period, with AAPC of −0.1% and −0.4% (*P* > 0.05), respectively. Among females, ASRs for lung cancer in 18 populations showed a significant increase during all over the period, with AAPC from 1.3% to 5.0% (*P* < 0.05; Table 2 and Fig. 1B).

In Asia, there were similar trends in ASRs for lung cancer for both males and females except for India (Tables 1 and 2 and Fig. 1). The increasing trends in Israel and Japan were observed from 1973 to 2007 (AAPC = 0.2% and 1.0% in males, 1.0% and 1.6% in females, respectively; *P* < 0.05), whereas decreasing trends in Singapore (AAPC = −1.5% in males, −0.7% in females, respectively; *P* < 0.05). ASRs for lung cancer in China (Hong Kong) and China (Shanghai) showed a stable trends from 1973 to 2007 (AAPC = −0.2% and −0.3% in males, −0.7% and 0.1% in females, respectively; *P* > 0.05).

**Figure 2:**
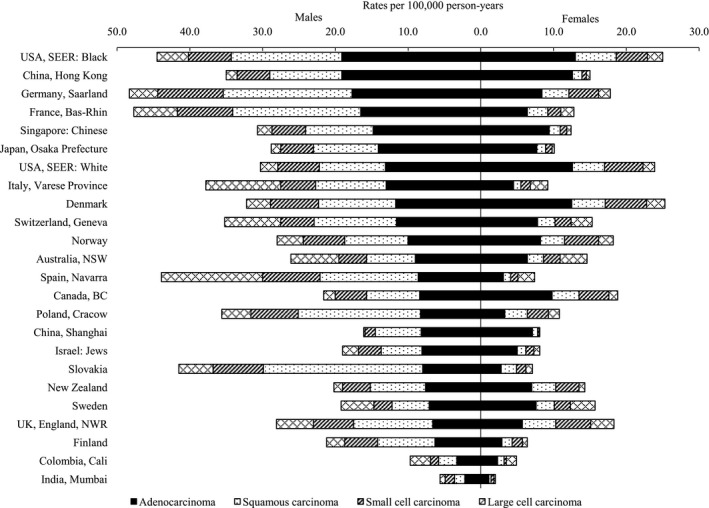
Age‐standardized lung cancer incidence rates by histologic subtypes for selected populations for the time period 2003–2007.

Age‐standardized incidence rates for lung cancer by histologic subtypes from 2003 to 2007 suggested that adenocarcinoma for males was the dominant histologic subtypes in 17 of 24 selected populations, whereas squamous carcinoma in the seven populations, and the main histologic subtypes for females were adenocarcinoma in all selected populations, followed by squamous carcinoma, small cell carcinoma, and large cell carcinoma (Fig. 2). The highest incidence of adenocarcinoma was observed in USA: Black (19.1 per 100,000 in males and 13.0 per 100,000 in females, respectively) and China (Hong Kong; 19.1 per 100,000 in males and 12.6 per 100,000 in females, respectively), and the lowest one was shown in India (2.2 per 100,000 in males and 1.1 per 100,000 in females, respectively). The squamous carcinoma was frequent histologic subtypes in the seven populations by males including Slovakia (21.9 per 100,000), Germany (17.7 per 100,000), France (17.6 per 100,000), Poland (16.8 per 100,000), Spain (13.5 per 100,000), UK, England (10.9 per 100,000), and Finland (7.9 per 100,000). The incidence of squamous carcinoma for females in the seven selected populations showed an extent from 1.0 per 100,000 to 4.6 per 100,000. The small cell carcinoma incidence rates for both males and females were higher than large cell carcinoma except for Spain (13.9 per 100,000 in males, 2.3 per 100,000 in females, respectively), Italy (10.3 per 100,000 in males, 2.4 per 100,000 in females, respectively), Australia (6.6 per 100,000, 3.7 per 100,000 in females, respectively), Sweden (4.5 per 100,000, 3.4 per 100,000 in females, respectively), and Colombia (2.8 per 100,000, 1.4 per 100,000 in females, respectively).

## Discussion

Age‐standardized incidence rates for lung cancer have been declining among males in most of Americas, Europe, and Oceania, while ASRs among females have been rising rapidly in these populations. Meanwhile, the increasing and decreasing trends were seen among males and females but no gender difference in Asian populations.

The overwhelming cause of lung cancer is tobacco smoking. The association between cigarette smoking and lung cancer was first established in 1950 20, and about 85% of lung cancer cases had a tobacco smoking history 21. Decreasing lung cancer incidence trends for males and increasing trends for females in some developed countries were observed which may be in part because of the variation of the prevalent smoking. Forey B et al. 22 suggested that the prevalence of tobacco smoking among males has declined since the 1960s in virtually all the areas except Spain, and incidence rate of lung cancer in Spain was continuing to increase. In our study, the increasing trend in Spain (Navarra) was observed across all the period. The reason of the phenomenon in Spain (Navarra) was not fully clear, but tobacco smoking might be a main reason. In several western countries including the United States, the United Kingdom, Canada, and Australia, the tobacco epidemic was established and peaked by the middle of the last century when lung cancer incidence rates had been decreasing in males and plateauing in females 23, 24. However, in our study, ASRs of lung cancer for females in USA: Black after 1988–1992 kept plateauing. This directly reflects the patterns of smoking prevalence among women in the past. These differences in smoking habits between North American and European women were observed. In North America, women started smoking earlier and consumed more cigarettes than in Frances 25. Smoking prevalence among women in the United States peaked at 33.3% in 1965 and remained at that level throughout the late 1970s. Since that time, the rate has declined to a level of 23.5% 21. Tobacco consumption started increasing in French women in the middle 1960s 26. The increasing trends of lung cancer incidence among females in European countries all over period were observed in our study. Besides smoking habit, the variation in lung cancer and its occurrence among females and males has been also hypothesized to be related to genetic susceptibility and biological indicators 27, 28, hormonal factors 29, and so on. Furthermore, the air pollution induced by industrialization in more developed countries is another possible cause. Traffic‐related No_x_ and CO showed significant correlations with the lung cancer incidence in females but not in males 30.

In Asia, the decreasing trends were observed in Singapore (Chinese) and India (Mumbai) males from 1973 to 2007 and China (Hong Kong) after mid‐1990s, although there was nonsignificant AAPC in China (Hong Kong) males and females. The declining trends for males in these populations can be explained by the gradual decline in smoking prevalence. However, the prevalence of smoking for females is low in Asia, which is different from western countries. It could reflect the effect of nonsmoking‐related risk factors more clearly. In Hong Kong, the prevalence of daily smokers decreased from 39.7% in 1982 to 22% in 2000 in males and from 5.6% to 3.5% in females 31. The other risk factors relating to lung cancer have changed especially indoor air pollution (IAP) including indoor fuel pollution and cooking. IAP is a major concern in less developed countries where biomass fuels are used for cooking and heating. Coal burning has been strongly associated with lung cancer risk in previous studies of rural and developing populations with high exposure 32, 33, and poor home ventilation can increase exposure to carcinogenic particulates which elevates lung cancer risk 34. The various possible etiologies of lung cancer have been probed in nonsmoking Chinese women living in Hong Kong 35. It is reported that the diet acts as an epidemiological confounder in the assessment of association between air pollution and female lung cancer in Hong Kong. Other risk factors for lung cancer including exposure to occupational and environmental carcinogens can also increase the risk of lung cancer 36. The lung cancer incidence rates for females in Shanghai kept stable in all period. Shanghai is a highly developed city, where the people's lifestyle is relatively close to the western developed countries. The pattern of lung cancer incidence may be likely because of the westernized lifestyle. Meanwhile, the use of rapeseed oil is common in Shanghai, which may be one of reasons. The association between lung cancer and frying pan fumes caused by rapeseed oil has been studied in Shanghai 37. A study from Shanghai had showed the stable level in lung cancer incidence for females and was likely to continue to increase in the future barring interventions were carried out for smoking cessation 38. In contrast, the lung cancer incidence in Japan and Israel (Jews) kept increasing trends. Lung cancer incidence reached a peak in the mid‐1990s for both males and females in Japan. As 70% of male lung cancer cases were reported because of active tobacco smoking 39, the observed peaks in lung cancer incidence might be a result of the high smoking prevalence in the 1960s or 1970s. The screening programs for lung cancer were implemented in 1987 40. Also, some changes in lifestyle, such as an increase in the proportion of dietary fat in energy intake 41, could be another explanation.

The occurrence of lung cancer is a slow process. The status of lung cancer resulted from different living environment and different way of life in the past 10 years or longer. With the decrease in the number of smoking, lung cancer incidence is still high. Besides exposure to occupational and environmental carcinogens, outdoor pollution needs to be focused 42. Since the 1970s, the United States 43 and Europe 44 have confirmed that lung cancer is associated with PM2.5. In October 2013, IARC classified particulate matter (PM) from outdoor air pollution as carcinogenic to humans and causes lung cancer 45. The surveillance of PM2.5 has begun in China, we cannot probe the relationship between the health problems and PM2.5 because of the shortage of data. Therefore, it is concluded that environmental pollution was one of an important risk factor. More than one‐half of the lung cancer deaths attributable to ambient fine particles were projected to have been in China and other East Asian countries 46.

This study has several strengths in that the data were abstracted from large, well‐established registries throughout the world. For the first time, data covering 35 years were analyzed to describe the variation of international trends in lung cancer incidence rates, which may stimulate further etiologic studies. In addition, this study can provide the update data of lung cancer for researchers in the world. This study, however, has limitations in that the trends in histologic subtypes of lung cancer were not investigated. Our study was also limited by the lack of nationwide cancer registries in some countries, where the registration data may not accurately reflect the true patterns in their nations.

In summary, our analysis on published CI5 data suggested that lung cancer incidence rates were declining among males and increasing among females in western populations where the highest incidence rate was still seen from 1973 to 2007. In Asia, there were increasing trends among males and females in some of countries and decreasing in the rest of populations. The international patterns of ASRs of lung cancer incidence were mainly attributed to tobacco smoking. However, other risk factors especially in females, including smoking habits, genetic susceptibility, and biological indicators, need to be further studied. The ongoing global tobacco reform is necessary to reduce the international burden of lung cancer.

## Conflict of Interest

The authors declare that they have no competing interests.

## Supporting information


**Figure S1.** The trends in age‐standardized smoking prevalence and ASRs of lung cancer for males.
**Figure S2.** The trends in age‐standardized smoking prevalence and ASRs of lung cancer for females.
**Figure S3.** The trends in mean annual consumption per capita and ASRs of lung cancer.
