# Supplementary material for: International trends in lung cancer incidence from 1973 to 2007
Source: Cancer Med. 2018 Mar 14;7(4):1479–89. doi: 10.1002/cam4.1359 (PMC5911623; doi:10.1002/cam4.1359)
Supplement: Supplementary file 1 — Figure S1. The trends in age‐standardized smoking prevalence and ASRs of lung cancer for males. Figure S2. The trends in age‐standardized smoking prevalence and ASRs of lung cancer for females. Figure S3. The trends in mean annual consumption per capita and ASRs of lung cancer. [file CAM4-7-1479-s001.pdf]

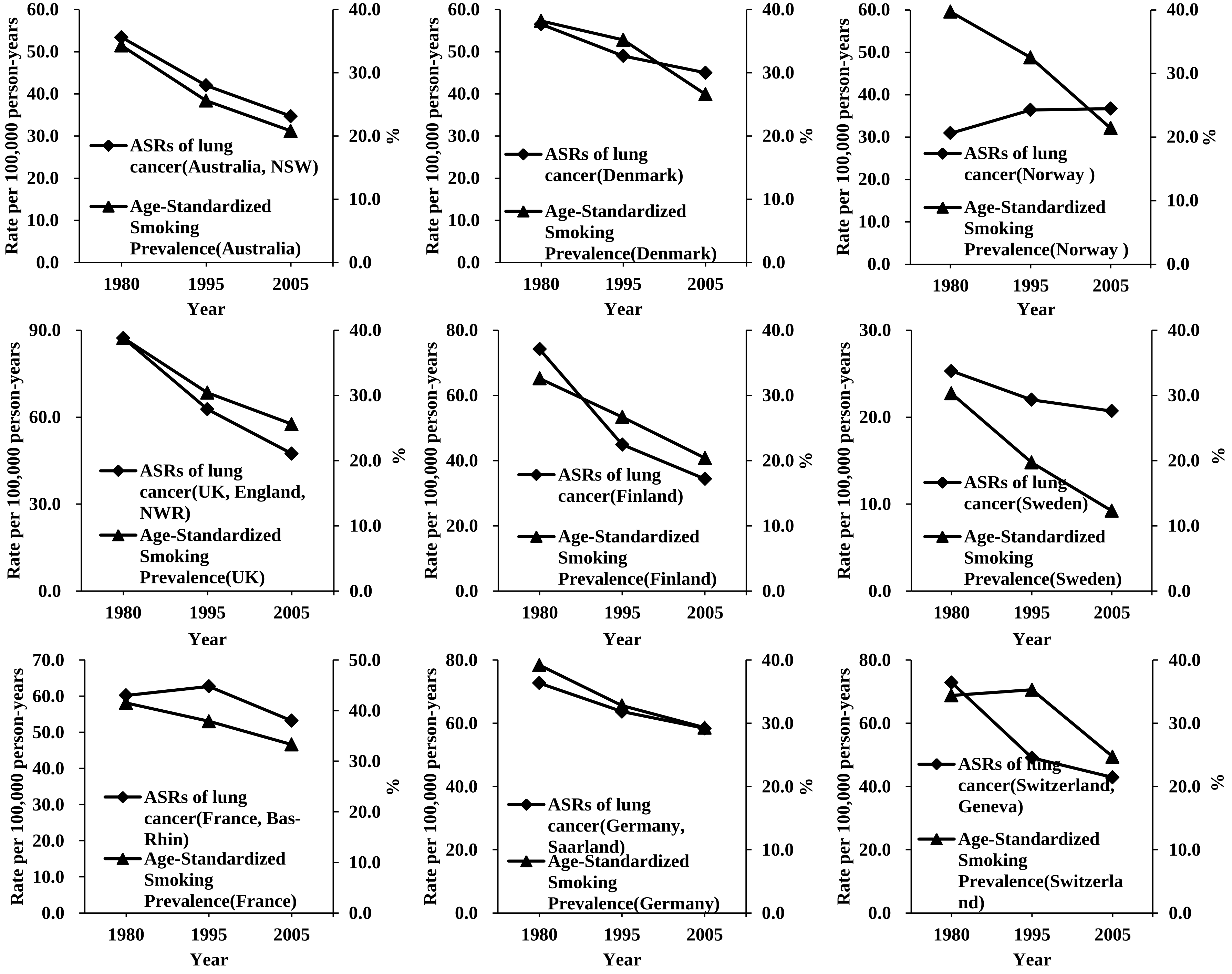

Rate per 100,000 person-years
%
Year
1980
1995
2005
ASRs of lung cancer(Australia, NSW)
Age-Standardized Smoking Prevalence(Australia)
ASRs of lung cancer(Denmark)
Age-Standardized Smoking Prevalence(Denmark)
ASRs of lung cancer(Norway)
Age-Standardized Smoking Prevalence(Norway)
ASRs of lung cancer(UK, England, NWR)
Age-Standardized Smoking Prevalence(UK)
ASRs of lung cancer(Finland)
Age-Standardized Smoking Prevalence(Finland)
ASRs of lung cancer(Sweden)
Age-Standardized Smoking Prevalence(Sweden)
ASRs of lung cancer(France, Bas-Rhin)
Age-Standardized Smoking Prevalence(France)
ASRs of lung cancer(Germany, Saarland)
Age-Standardized Smoking Prevalence(Germany)
ASRs of lung cancer(Switzerland, Geneva)
Age-Standardized Smoking Prevalence(Switzerland)

Continued

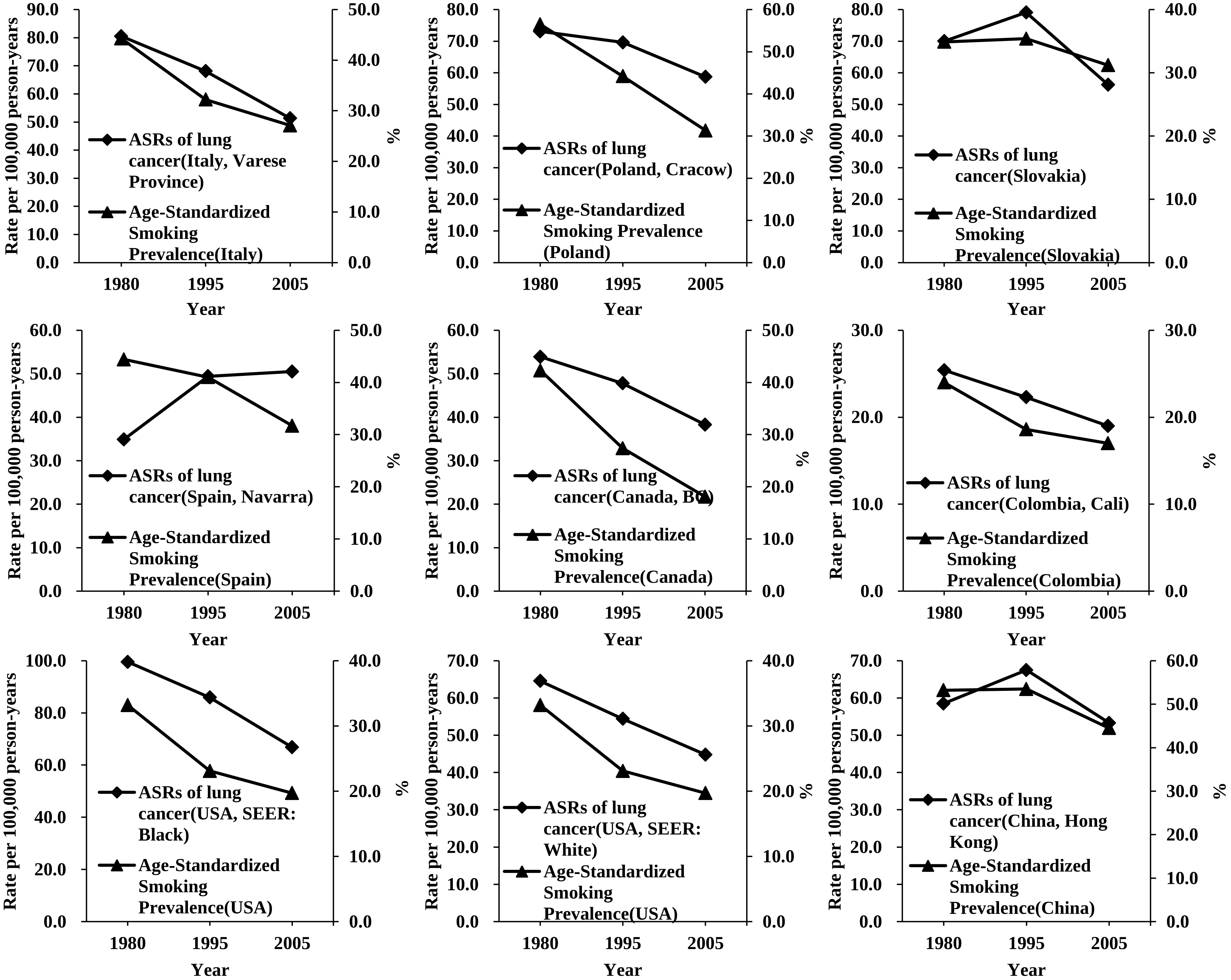
Rate per 100,000 person-years
%
Year
1980
1995
2005
ASRs of lung cancer(Italy, Varese Province)
Age-Standardized Smoking Prevalence(Italy)
ASRs of lung cancer(Poland, Cracow)
Age-Standardized Smoking Prevalence (Poland)
ASRs of lung cancer(Slovakia)
Age-Standardized Smoking Prevalence(Slovakia)
ASRs of lung cancer(Spain, Navarra)
Age-Standardized Smoking Prevalence(Spain)
ASRs of lung cancer(Canada, BC)
Age-Standardized Smoking Prevalence(Canada)
ASRs of lung cancer(Colombia, Cali)
Age-Standardized Smoking Prevalence(Colombia)
ASRs of lung cancer(USA, SEER: Black)
Age-Standardized Smoking Prevalence(USA)
ASRs of lung cancer(USA, SEER: White)
Age-Standardized Smoking Prevalence(USA)
ASRs of lung cancer(China, Hong Kong)
Age-Standardized Smoking Prevalence(China)

Continued

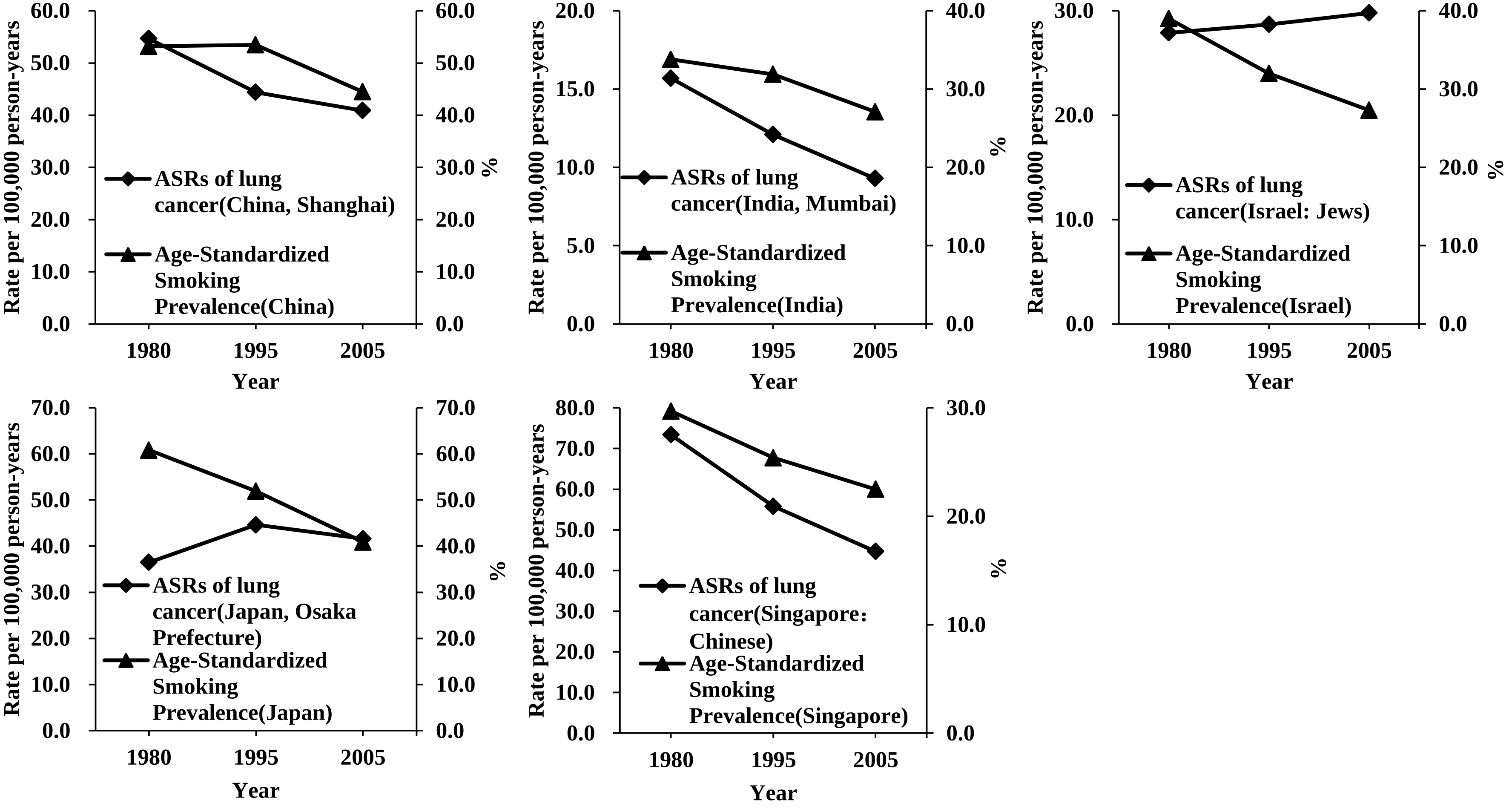

Figure 1 The trends in Age-Standardized smoking prevalence and ASRs of lung cancer for males

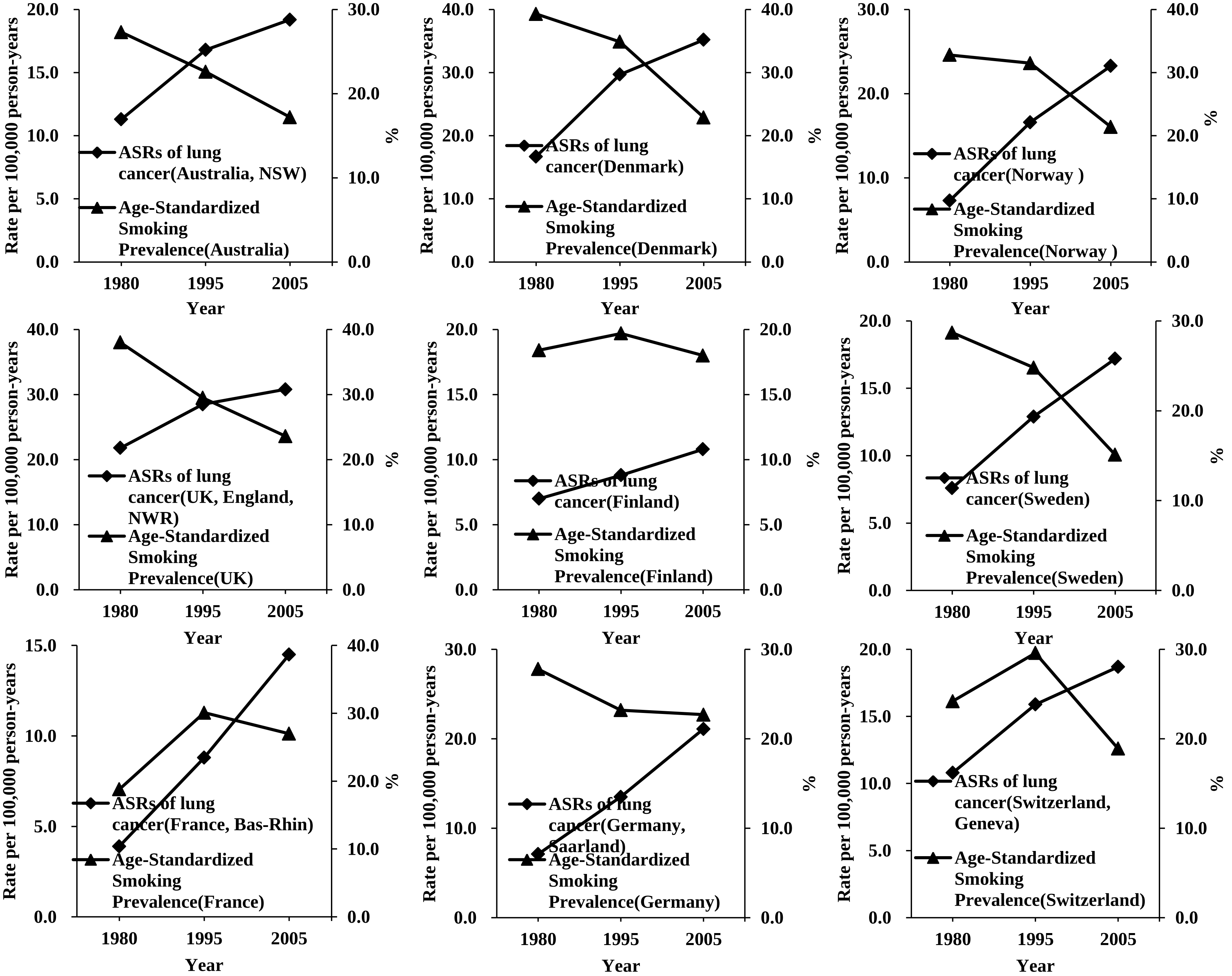

Rate per 100,000 person-years
%
Year
1980
1995
2005
ASRs of lung cancer(Australia, NSW)
Age-Standardized Smoking Prevalence(Australia)
ASRs of lung cancer(Denmark)
Age-Standardized Smoking Prevalence(Denmark)
ASRs of lung cancer(Norway)
Age-Standardized Smoking Prevalence(Norway)
ASRs of lung cancer(UK, England, NWR)
Age-Standardized Smoking Prevalence(UK)
ASRs of lung cancer(Finland)
Age-Standardized Smoking Prevalence(Finland)
ASRs of lung cancer(Sweden)
Age-Standardized Smoking Prevalence(Sweden)
ASRs of lung cancer(France, Bas-Rhin)
Age-Standardized Smoking Prevalence(France)
ASRs of lung cancer(Germany, Saarland)
Age-Standardized Smoking Prevalence(Germany)
ASRs of lung cancer(Switzerland, Geneva)
Age-Standardized Smoking Prevalence(Switzerland)

Continued

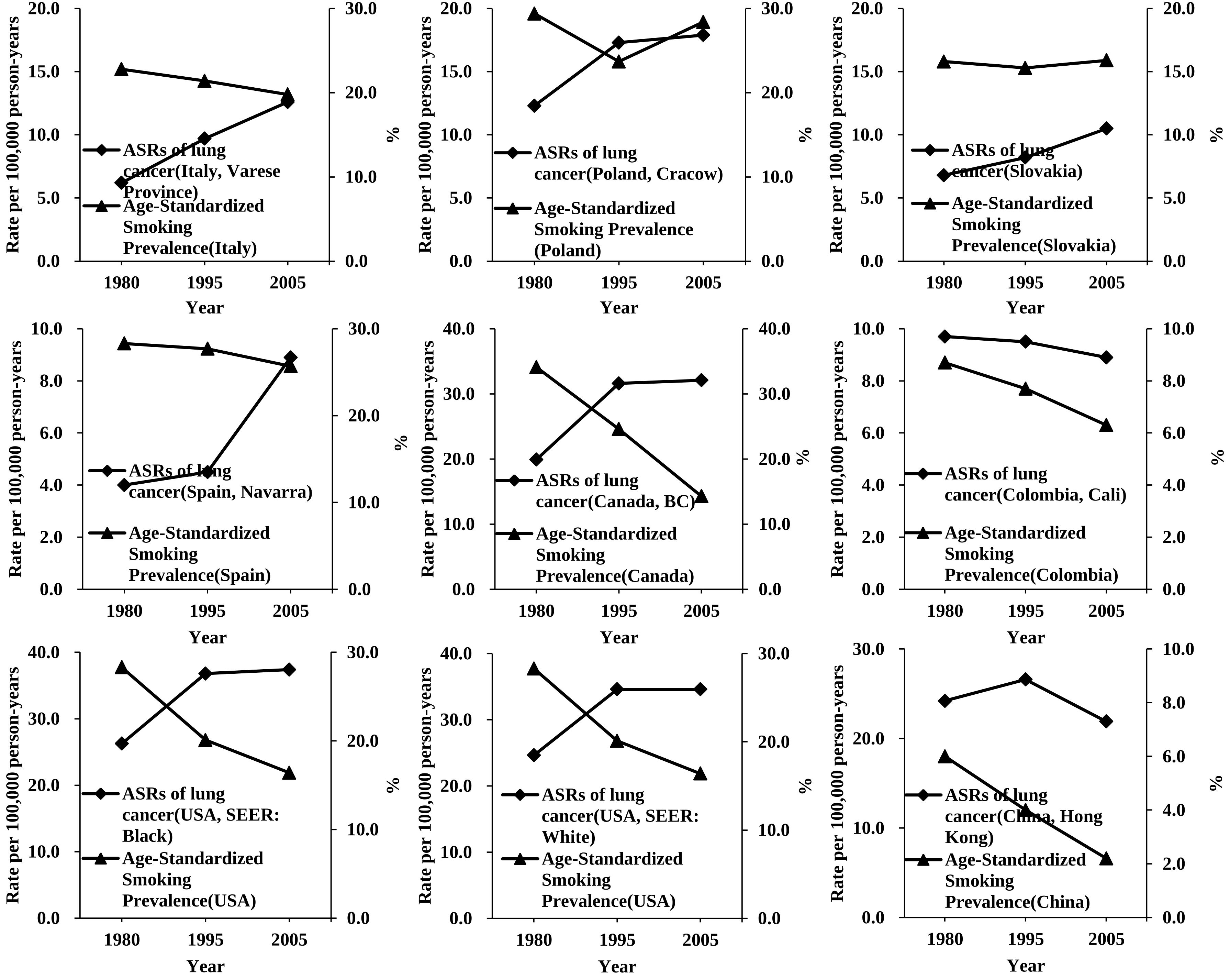

Rate per 100,000 person-years
%
Year
1980
1995
2005
ASRs of lung cancer(Italy, Varese Province)
Age-Standardized Smoking Prevalence(Italy)
ASRs of lung cancer(Poland, Cracow)
Age-Standardized Smoking Prevalence (Poland)
ASRs of lung cancer(Slovakia)
Age-Standardized Smoking Prevalence(Slovakia)
ASRs of lung cancer(Spain, Navarra)
Age-Standardized Smoking Prevalence(Spain)
ASRs of lung cancer(Canada, BC)
Age-Standardized Smoking Prevalence(Canada)
ASRs of lung cancer(Colombia, Cali)
Age-Standardized Smoking Prevalence(Colombia)
ASRs of lung cancer(USA, SEER: Black)
Age-Standardized Smoking Prevalence(USA)
ASRs of lung cancer(USA, SEER: White)
Age-Standardized Smoking Prevalence(USA)
ASRs of lung cancer(China, Hong Kong)
Age-Standardized Smoking Prevalence(China)

Continued

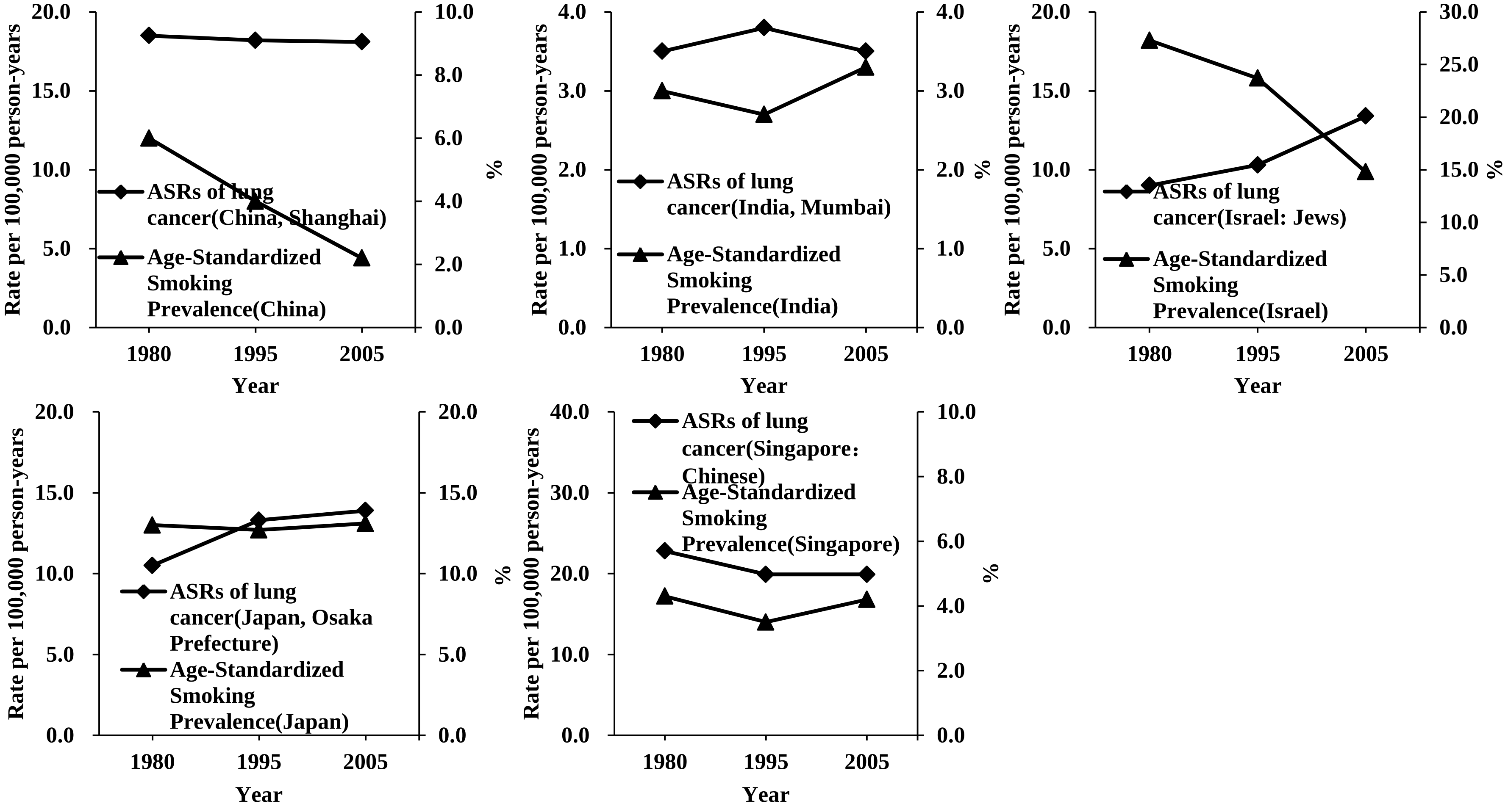

Figure 2 The trends in Age-Standardized smoking prevalence and ASRs of lung cancer for females

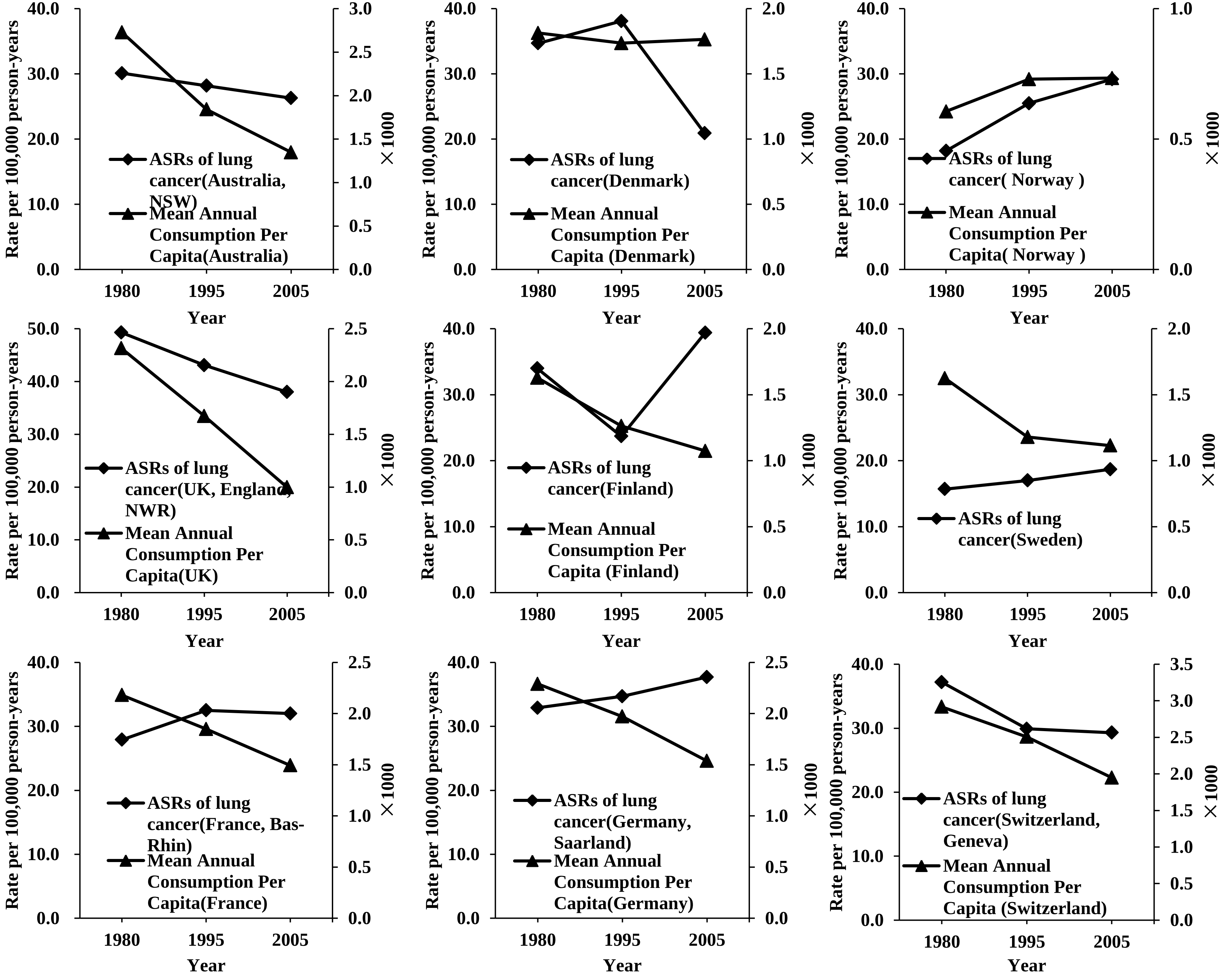
Rate per 100,000 person-years
Year
×1000
1980
1995
2005
ASRs of lung cancer(Australia, NSW)
Mean Annual Consumption Per Capita(Australia)
ASRs of lung cancer(Denmark)
Mean Annual Consumption Per Capita (Denmark)
ASRs of lung cancer(Norway)
Mean Annual Consumption Per Capita(Norway)
ASRs of lung cancer(UK, England, NWR)
Mean Annual Consumption Per Capita(UK)
ASRs of lung cancer(Finland)
Mean Annual Consumption Per Capita (Finland)
ASRs of lung cancer(Sweden)
ASRs of lung cancer(France, Bas-Rhin)
Mean Annual Consumption Per Capita(France)
ASRs of lung cancer(Germany, Saarland)
Mean Annual Consumption Per Capita(Germany)
ASRs of lung cancer(Switzerland, Geneva)
Mean Annual Consumption Per Capita (Switzerland)

Continued

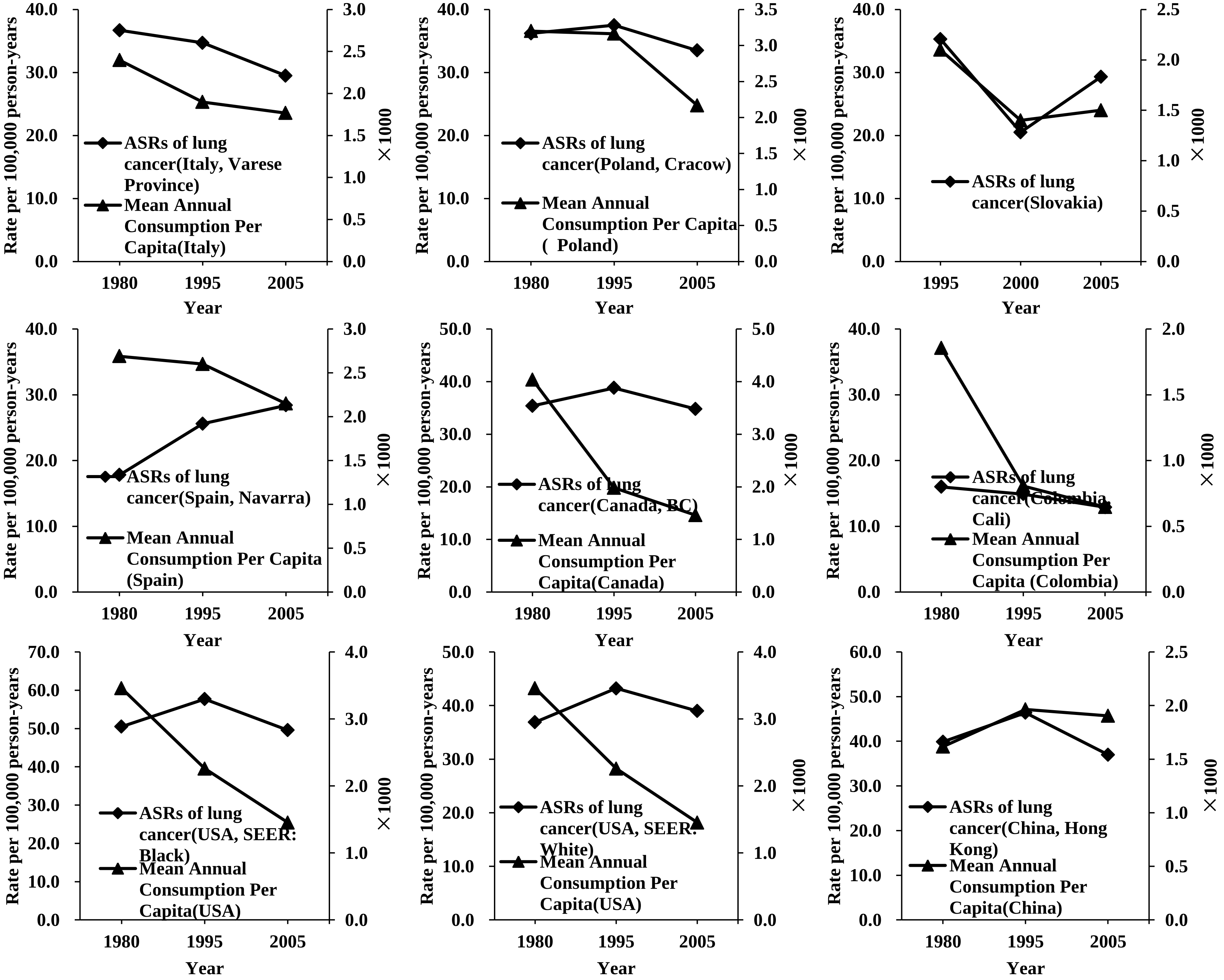

Rate per 100,000 person-years
Year
×1000
ASRs of lung cancer(Italy, Varese Province)
Mean Annual Consumption Per Capita(Italy)
ASRs of lung cancer(Poland, Cracow)
Mean Annual Consumption Per Capita (Poland)
ASRs of lung cancer(Slovakia)
ASRs of lung cancer(Spain, Navarra)
Mean Annual Consumption Per Capita (Spain)
ASRs of lung cancer(Canada, BC)
Mean Annual Consumption Per Capita(Canada)
ASRs of lung cancer(Colombia, Cali)
Mean Annual Consumption Per Capita (Colombia)
ASRs of lung cancer(USA, SEER: Black)
Mean Annual Consumption Per Capita(USA)
ASRs of lung cancer(USA, SEER: White)
Mean Annual Consumption Per Capita(USA)
ASRs of lung cancer(China, Hong Kong)
Mean Annual Consumption Per Capita(China)

Continued

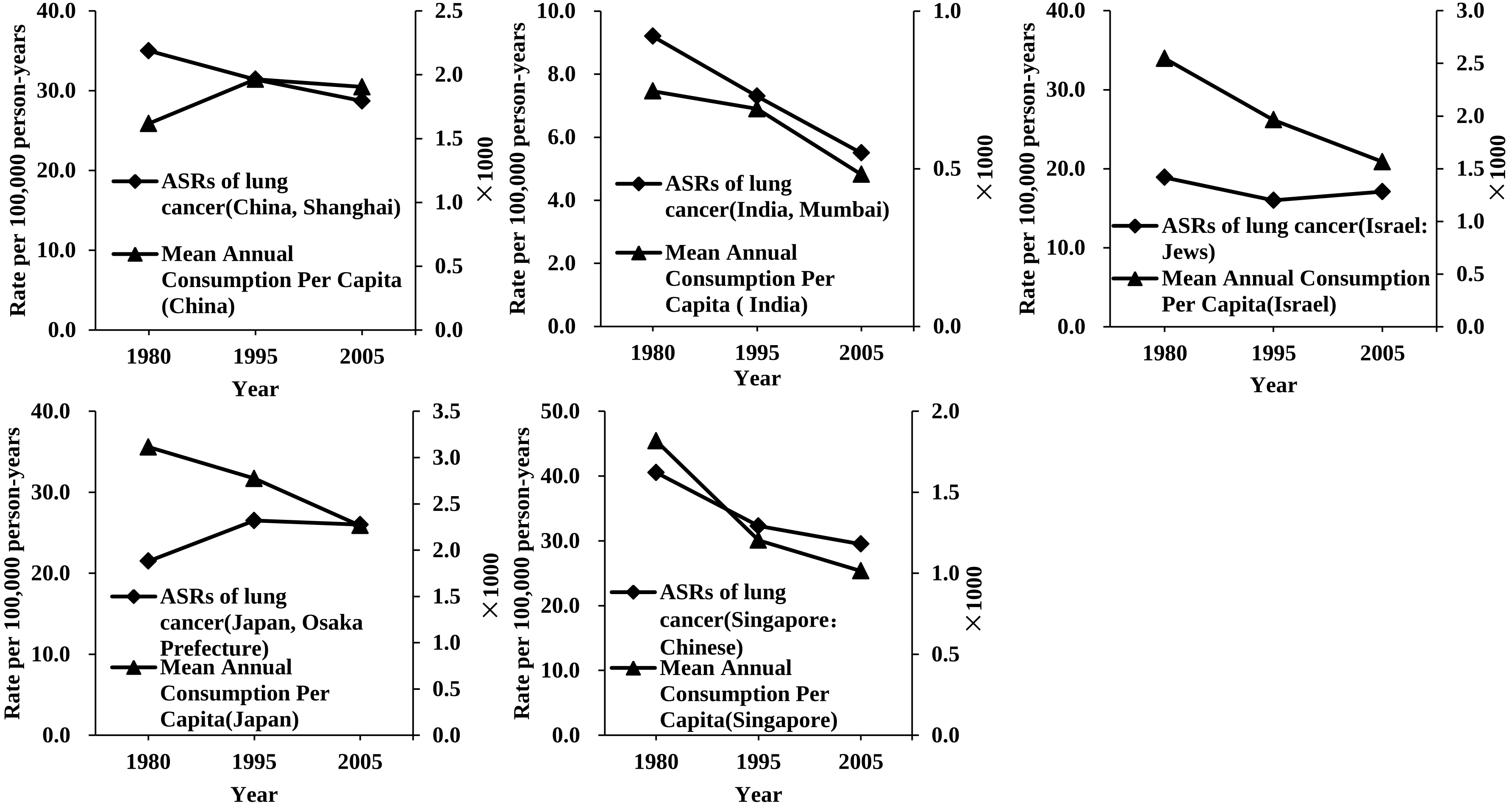

Figure 3 The trends in mean annual consumption per capita and ASRs of lung cancer
